# Insights into the diverse mechanisms and effects of variant *CUL3*-induced familial hyperkalemic hypertension

**DOI:** 10.1186/s12964-023-01269-z

**Published:** 2023-10-16

**Authors:** Prashant Sharma, Harish E. Chatrathi

**Affiliations:** 1 NIH Undiagnosed Diseases Program, Common Fund, Office of the Director, National Institutes of Health, Bethesda, MD USA; 2 College of Medicine, University of Arkansas for Medical Sciences, Little Rock, AR USA

**Keywords:** CUL3, Hyperkalemia, Hypertension, Post-translational modifications, Substrate-adaptor binding

## Abstract

Familial hyperkalemic hypertension (FHHt), also known as Pseudohypoaldosteronism type II (PHAII) or Gordon syndrome is a rare Mendelian disease classically characterized by hyperkalemia, hyperchloremic metabolic acidosis, and high systolic blood pressure. The most severe form of the disease is caused by autosomal dominant variants in *CUL3* (Cullin 3), a critical subunit of the multimeric CUL3-RING ubiquitin ligase complex. The recent identification of a novel FHHt disease variant of *CUL3* revealed intricacies within the underlying disease mechanism. When combined with studies on canonical *CUL3* variant-induced FHHt, these findings further support CUL3’s role in regulating renal electrolyte transport and maintaining systemic vascular tone. However, the pathophysiological effects of *CUL3* variants are often accompanied by diverse systemic disturbances in addition to classical FHHt symptoms. Recent global proteomic analyses provide a rationale for these systemic disturbances, paving the way for future mechanistic studies to reveal how *CUL3* variants dysregulate processes outside of the renovascular axis.

Video Abstract

**Supplementary Information:**

The online version contains supplementary material available at 10.1186/s12964-023-01269-z.

## Background

**Fig. 1:**
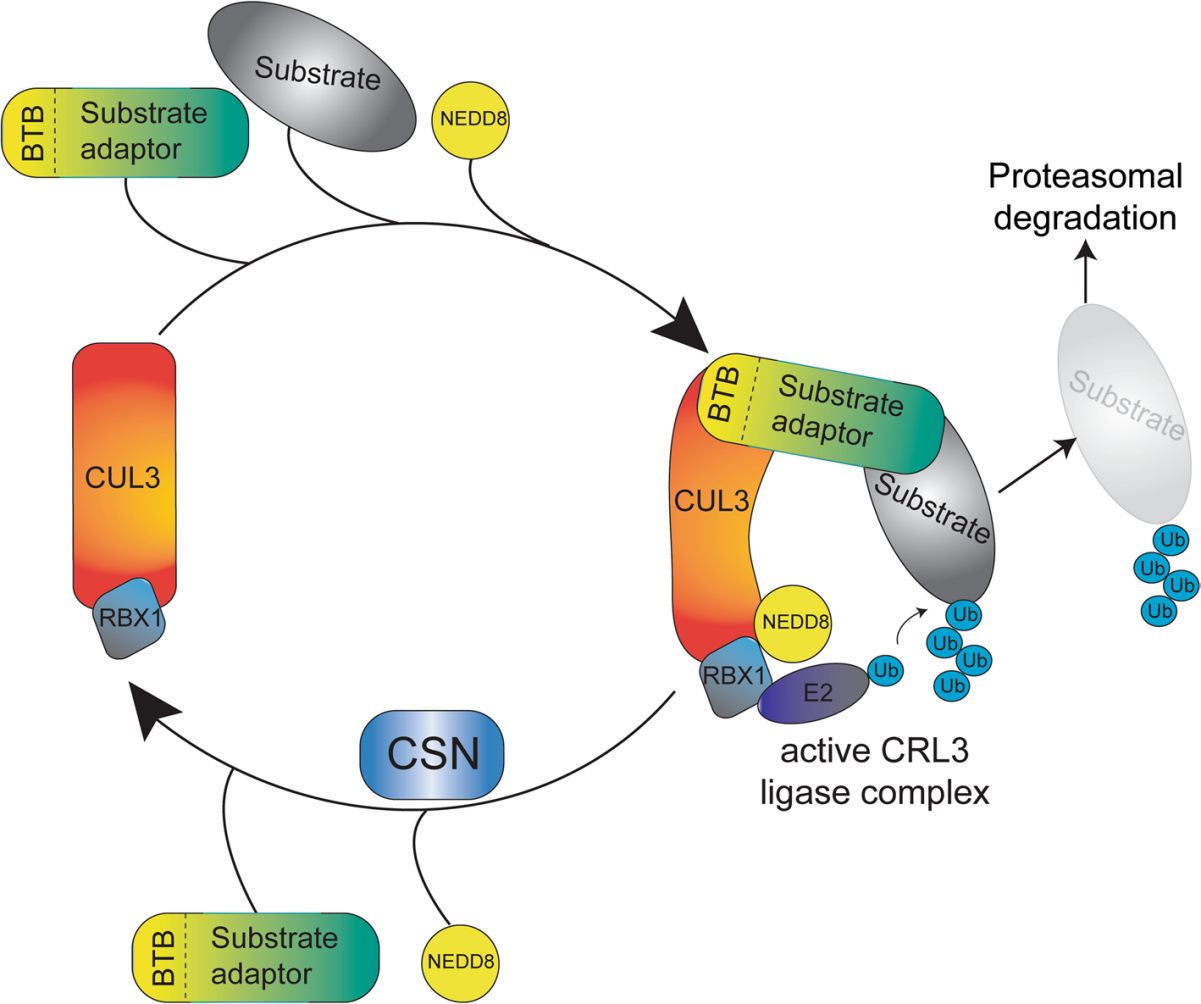
The neddylation-deneddylation cycle regulates the Cullin 3 ligase (CRL3) activity. The RING-box protein RBX1 tightly associates at the C-terminus of the CUL3 protein and forms the catalytic core of the CRL3 ligase complex. The CUL3 protein acts as a scaffold and interacts at the N-terminus with the BTB domain-containing substrate adaptor, which recruits target proteins for ubiquitination and subsequent degradation in the proteasome. The covalent modification of CUL3 with NEDD8 (neddylation) activates the ligase complex by facilitating the efficient transfer of ubiquitin to the substrate. Multi-subunit enzyme complex CSN removes NEDD8 (deneddylation) and deactivates the active ligase complex

Familial hyperkalemic hypertension (FHHt, also known as Pseudohypoaldosteronism type II or Gordon Syndrome) comprises the clinical manifestation of overactivated Na^+^-Cl^−^-Cotransporter (NCC) in the distal convoluted tubule (DCT) of the renal nephron. The classical clinical phenotype of FHHt includes metabolic acidosis, hyperkalemia, hyperchloremia, and arterial hypertension. The clinical picture of FHHt is the mirror presentation of Gitelman syndrome (OMIM:263800), which includes metabolic alkalosis, hypokalemia, hypocalciuria, and arterial hypotension [1]. Gitelman syndrome is associated with loss-of-function variants in the *SLC12A3* gene encoding the NCC [2]. To date, no disease-causing genetic variant in the *SLC12A3 *gene has been identified in FHHt patients. The overactivation of the NCC in FHHt patients is caused by the biochemical dysregulation of the WNK-SPAK/OSR1 signaling cascade resulting in increased phosphorylation and overactivation of NCC [3–5]. The WNK protein abundance/activity is controlled by a RING domain-containing E3 ubiquitin ligase complex CRL3 (CUL3-RING ligase 3). The functional CRL3 complex is formed by the Cullin 3 (CUL3) protein that acts as a scaffold to interact at the N-terminus with BTB domain-containing substrate adaptor protein and at the C-terminus with RBX1 protein for ubiquitination of substrate proteins [6–8]. The activity of the CRL3 complex is regulated by reversible post-translational modification of CUL3 with NEDD8 (neddylation) [9]. Removal of NEDD8 (deneddylation) turns off the active ligase and is carried out by a multi-subunit enzyme complex CSN [10] (Fig. 1). In the DCT, CUL3 interacts with a substrate adaptor KLHL3 (kelch-like-family-member-3) to target WNKs for ubiquitination and subsequent proteasomal degradation [11–13].

Genetic variants have been identified in *CUL3*, *KLHL3*, *WNK1*, and *WNK4* in FHHt patients, suggesting a genotype–phenotype correlation [14–17]. The age of onset and the degree of disease severity is correlated with underlying genetic variants. Variants in *CUL3* are often associated with growth impairment in addition to hypertension and electrolyte imbalances and are commonly manifested in infants and children [14, 18–21], while variants in *KLHL3* and *WNKs* are associated with hypertension and electrolyte imbalances in mostly adult FHHt patients [14–16]. All known *CUL3* variants causing FHHt are inherited in an autosomal dominant manner. The canonical *CUL3* variant is a 57 amino acid deletion (aa 403–459) resulting from exon 9 skipping of mRNA (CUL3Δ9; also known as CUL3Δ403–459). Recently, our group reported a novel de novo variant in *CUL3* (NM_003590.5: c.1420_1431del12; Phe474_Met477del) in a pediatric FHHt patient [21]. The biochemical characterization of this novel *CUL3* variant in the FHHt patient’s urinary exosome vesicles (uEVs) and cells established the deletion of amino acids 474–477 in exon 10 of CUL3 as the source of electrolyte imbalance and hypertension in vivo [21]. A detailed clinical evaluation of our patient revealed multiple anomalies in addition to FHHt symptoms, which raises the possibility that FHHt-causing *CUL3* variants exert widespread pathophysiological effects. In this review, we describe the role of *CUL3* variants in electrolyte/metabolic imbalances and hypertension in FHHt. We reviewed clinical phenotypes of FHHt patients associated with *CUL3* variants and discuss the possible consequences of dysregulated CRL3 complexes in the pathophysiology of other FHHt-associated disorders. We provide a rationale for wide-spectrum developmental defects associated with *CUL3* variants and suggest that the classical definition of FHHt as a ‘renal-specific disease’ needs further scrutiny.

### The NCC signaling pathway genes regulate electrolyte balance and blood pressure

Renal Na^+^ reabsorption contributes to long-term arterial blood pressure regulation by affecting plasma volume [22]. Inherited monogenic disorders that result in congenital forms of hypertension are consequences of genetic variants that either directly encode renal salt transporters or modulate their activities. Na^+^ reabsorption in the nephron is primarily accomplished by the proximal tubules, while distal tubules mediate electrolyte fine-tuning in response to variable dietary intake [23]. The distal convoluted tubule (DCT) of the nephron expresses NCC at the apical membrane. The connecting tubules (CNT) connect the collecting duct (CD), which contains epithelial Na^+^ channels (ENaC), renal outer medullary K^+^ channels (ROMK), and flow-dependent large Ca^2+^-activated K^+^ channels (BK). The NCC mediates Na^+^ reabsorption in the DCT, but the activity of NCC influences K^+^ secretion by ROMK and BK channels in the subsequent CD segment of the distal nephron. The activation and abundance of NCC are reduced when serum K^+^ levels are elevated. This increases Na^+^ delivery to ENaC in sequential distal segments, enhancing electrogenic K^+^ secretion via ROMK and BK channels and restoring serum K^+^ levels. Conversely, during low plasma K^+^ levels, NCC activation and abundance are strongly increased in the DCT, limiting Na^+^ delivery to downstream segments.

In the absence of a known genetic linkage between FHHt and the NCC gene (*SLC12A3*), it is plausible that the regulation of NCC activity and abundance is solely carried out by the upstream signaling pathway that regulates NCC phosphorylation and dephosphorylation. Two serine-threonine kinases, SPAK and OSR1, phosphorylate NCC on three residues, i.e., Thr46, Thr55, and Thr60 at the N-terminal tail [3]. In vitro, both SPAK and OSR1 are equally able to phosphorylate NCC*.* Loss-of-function *Spak* mouse models show a dramatic reduction in NCC phosphorylation and display the salt-wasting phenotype observed in human Gitelman syndrome [5, 24, 25]. Moreover, constitutive activation of SPAK in the early DCT, concurrent with NCC hyperphosphorylation, recapitulates the FHHt phenotype [26]. Genome-wide association studies identified a highly conserved intronic element within the *STK39* gene, which is responsible for increased expression of SPAK and higher blood pressure in humans [27]. These findings are consistent with the concept that SPAK activity is essential for NCC phosphorylation in vivo. The global depletion of OSR1 is embryonically lethal; the targeted disruption of OSR1 in the distal nephron in mice leads to an increased abundance of pNCC, likely to compensate for the reduced phosphorylation and activity of OSR1’s main target, NKCC2 [28].

The activity of SPAK/OSR1 itself is also regulated by phosphorylation mediated by the upstream lysine-deficient protein kinases WNKs (with no-lysine [K] kinases). The four distinct isoforms of the WNK family (WNK1, WNK2, WNK3, and WNK4) in mammals possess an N-terminal kinase domain but lack the conserved catalytic lysine residue in subdomain II, which is required for ATP binding [29, 30]. The crystal structure of WNK1 revealed that the catalytic lysine residue in subdomain I mediates the kinase activity and accounts for the missing lysine in subdomain II [31]. Two isoforms of WNKs (WNK1 and WNK4) have been shown to phosphorylate and activate both SPAK and OSR1 within the T-loop of the catalytic domain (Thr233 in SPAK and Thr185 in OSR1) and a serine residue located within a C-terminal non-catalytic region (Ser373 in SPAK and Ser325 in OSR1) [32]. Both WNK1 and WNK4 are expressed in the kidney. Notably, a splice variant of WNK1 that lacks the entire N-terminal kinase domain (KS-WNK1) is primarily expressed in the DCT [33, 34]. Interestingly, in response to changes in the extracellular potassium, KS-WNK1 plays a critical role in the formation of large puncta (WNK bodies) in the DCT. These puncta contain WNK-SPAK/OSR1 pathway components, and the formation of these puncta is a hallmark of WNK activation [35]. In addition, KS-WNK1 has also been shown to directly activate the WNK4 isoform [36]. The pathological role of WNKs in electrolyte imbalance and hypertension was discovered by the identification of disease-associated variants in the *WNK1 *and* WNK4* genes [16]. The intronic deletions in *WNK1* lead to ectopic expression of full-length WNK1 [16]. Consistent with this notion, the mouse model demonstrated that overexpression of WNK1 enhances NCC phosphorylation/activation, increasing Na^+^ resorption and raising blood pressure [37]. Conversely, *Wnk1* heterozygous mice displayed a significant decrease in blood pressure, while complete deletion of *Wnk1* is embryonically lethal, suggesting WNK1’s critical role in development [38]. The PHAII-associated missense variants (Glu562Lys, Asp564Ala, and Gln565Glu) in WNK4 are clustered within the conserved acidic domain [16]. Mice deficient in WNK4 displayed reduced phosphorylation/activation of NCC and normal blood pressure, recapitulating a mild Gitelman syndrome phenotype [39]. Knock-in mice carrying a heterozygous Asp561Ala (D561A) missense variant (*Wnk4* ^D561A/+^), corresponding to the *WNK4*^D564A/+^ human PHAII causing variant, exhibited the canonical PHAII phenotypes of high blood pressure, hyperkalemia, and metabolic acidosis. These *Wnk4* ^D561A/+^ mice also showed increased phosphorylation of NCC, OSR1 and SPAK kinases [40]. The canonical PHAII phenotypes in *Wnk4* ^D561A/+^ mice were corrected by mating with mice lacking phosphorylation of SPAK and OSR1 (*Wnk4*^D561A/+^ *Spak* ^T243A/T243A^ *Osr1*^T185A/+^ triple knock-in mice). Moreover, the triple knock-in mice showed abrogation of increased NCC phosphorylation [41]. These studies clearly demonstrate that NCC phosphorylation is dependent on the WNK-SPAK/OSR1 signaling cascade in the kidney and highlight the essential role of this cascade in maintaining electrolyte homeostasis and blood pressure. Contrary to phosphorylation, dephosphorylation inactivates the NCC pathway and is mediated by protein phosphatases (PPs) [42]. The physiological role of PPs in NCC regulation requires further research.

The levels of WNK protein are regulated through an E3 ubiquitin ligase complex, CUL3-KLHL3. Within this complex, CUL3 acts as a protein scaffold for KLHL3, and KLHL3 serves as a substrate-binding adaptor for ubiquitination and subsequent proteasomal degradation of WNKs. The identification of disease-associated variants in both *CUL3* and *KLHL3* in PHAII patients highlights the important role of the CUL3-KLHL3 complex in the pathophysiology of electrolyte imbalance and hypertension [14, 21, 43]. In fact, the FHHt symptoms associated with both *CUL3 *and* KLHL3* variants are considered more severe than FHHt symptoms associated with variants in WNKs, suggesting a genotype–phenotype correlation [14, 17, 43]. This is likely because CUL3/KLHL3 variants are impaired in WNK degradation and retained an overall higher WNK level than WNK variants affecting only one allele, which likely results in the discrepancy in disease severity.

The KLHL3 protein is composed of BTB and BACK domains on the N-terminal, as well as 5–6 bladed kelch motifs (repeats) at the C-terminal. The BTB domain binds to CUL3, and Kelch repeats bind substrate WNK1 and WNK4 [11, 12]. The prevalence of FHHt-associated variants found in *KLHL3* is higher than *WNK1*, *WNK4*, and *CUL3* variants [17]. FHHt variants exist in all three domains of KLHL3 and are inherited in both dominant and recessive fashions [17, 43]. Variants in BTB and BACK domains abrogate interaction with CUL3, while variants clustered within or ‘nearby’ kelch repeats impair the ability of KLHL3 to interact with WNK1 and WNK4 [11–13]. Shibata et al. showed that the S433 residue, located within the Kelch domain, is frequently mutated (S433N) in FHHt patients [14, 43] and is a site for phosphorylation by protein kinase C (PKC) in response to angiotensin II signaling, which increases arterial tone and promotes renal sodium absorption. Mechanistically, the authors showed that phosphorylation of KLHL3 at serine 433 (S433) prevents WNK4 binding and ultimately decreases its proteasomal degradation [44].

Complete deletion of *klhl3* in mice (*klhl3*^−/−^) leads to PHAII-like phenotypes, while heterozygous (*klhl3*^+/-^) mice fail to show a PHAII disease phenotype, suggesting that a dominant negative effect of *KLHL3* variant is responsible for the disease phenotype [45]. To investigate the pathogenesis of PHAII caused by autosomal dominant *KLHL3* variants, Susa et al.generated and analyzed *Klhl3*^R528H/+^ knock-in mice. *Klhl3*^R528H/+^ knock-in mice showed an increased abundance of WNK1, WNK4, and phosphorylated NCC in mouse kidney samples while exhibiting classical FHHt phenotypes of salt-sensitive hypertension, hyperkalemia, and metabolic acidosis. Using fluorescence correlation spectroscopy, the authors showed that the R528H KLHL3 variant lost interaction with WNK1 and WNK4. This results in impaired KLHL3-CUL3 mediated ubiquitination of WNKs and increased WNK-SPAK/OSR1-NCC signaling in the *Klhl3*^R528H/+^ mouse kidney [46]. To further define the role of the specific WNK isoform, Susa and colleagues also generated *Wnk4*^**−/−**^*Klhl3*^R528H/+^ and *Wnk4*^**−/−**^*Klhl3*^R528H/R528H^ mice and showed markedly increased WNK1 levels in kidney samples. However, phosphorylation of SPAK and NCC and total NCC expression were reduced to undetectable levels in the distal convoluted tubules of these mice. The authors conclude that despite increased WNK1 levels, WNK4 dysregulation is crucial for the onset of FHHt [47]. KS-WNK1, which is the major isoform expressed in DCT, was not evaluated in the mutant mice.

FHHt-causing variants in the *CUL3* gene are autosomal dominant and lead to the most severe form of the disease. The canonical *CUL3* variants cause skipping of exon 9 (CUL3Δ9), resulting in a deletion of 57 amino acids (Δ403–459) within the 4HB (4-helix bundle) domain of CUL3 [14]. Recently, our group identified a novel de novo variant in exon 10 of *CUL3* in a pediatric FHHt patient. This variant results in the deletion of 4 amino acids (Δ474-477) within the αβ1 domain of CUL3 [21]. This patient displayed the classical FHHt phenotype and other notable congenital anomalies (see below), as well as increased phosphorylation of SPAK/OSR1 and NCC in patient-derived urinary extracellular vesicles (uEV). Moreover, patient-derived dermal fibroblast showed a reduced abundance of total CUL3, hyperneddylation, increased CUL3-KLHL3 binding, and increased levels of WNK4. While fibroblasts may not be the most direct model system to demonstrate renal effects, the successful demonstration of molecular consequences of CUL3Δ474-477 in dermal fibroblasts, which are easily accessible and can be grown upon culturing, may allow future studies to use these patient-derived cells to study the consequences of FHHt causing CUL3 variants, given the limitation in obtaining patient’s DCT cells.

Treatment of the patient with thiazide diuretics corrected both hyperkalemia and hypertension, which provides evidence of increased signaling through the WNK4-SPAK/OSR1-NCC axis in FHHt [21].

The pathophysiology of CUL3Δ403–459 variant is well-studied in various mouse models (Table 1). The complete deletion of *Cul3* in mice is embryonically lethal [48], exemplifying the essential role that CUL3 plays in development. Like heterozygous *Klhl3* mice, *Cul3* heterozygotes do not manifest with FHHt phenotypes [45, 49], suggesting that CUL3Δ9 exerts a dominant-negative effect. It is also notable that *Cul3* and *Klhl3* double heterozygous mice (*Cul3*^+/-^ *Klhl3*^+/-^), generated by the renal tubule-specific system, showed increased abundance of WNK4, pSPAK, pNCC,and total NCC compared to control mice. *Cul3*^+/-^ *Klhl3*^+/-^ mice displayed increased plasma potassium (K^+^) but no change in the systolic blood pressure (SBP). The change in SBP was only significantly different when *Cul3*^+/-^ *Klhl3*^+/-^ mice were fed a high-sodium/normal-potassium diet [50]. These findings suggest that the development of severe hypertension and other systemic disorders are likely the results of the extra-renal effects of FHHt-causing *CUL3* variants.

**Table 1:** List of published *Cul3* mouse models and main findings relevant to FHHt

Publication	Genotype	Main findings
McCormick el al*.* (2014) [51]	KS-*Cul3*^−/−^ (Kidney specific *Cul3* deletion)	Increased protein abundance of WNK kinases and pNCC but no change in KLHL3 abundance. Hypochloremic alkalosis, mild hypokalemia, salt-sensitive hypotension after long term *Cul3* deletion. Kidney damage characterized by inflammation and fibrosis
Schumacher et al. (2015) [52]	*Cul3*^+/Δ403–459^	Homozygous embryonic lethal. Increased protein abundance of WNK4, pSPAK, and pNCC in *Cul3*^+/Δ403–459^ kidney lysates. No change in KLHL3 abundance. Reduced CUL3Δ403–459 compared to WT-CUL3. Hyperkalaemia, hyperchloraemia, metabolic acidosis, and high blood pressure. Lower body weight and body length of *Cul3*^+/Δ403–459^ compared to *Cul3*^+/+^
Araki et al.(2015) [53]	*Cul3*^G(−1)A/+^ & *Cul3*^T(−6)G/T(−6)G^	Homozygous *Cul3*^G(−1)A/+^ embryonic lethal. No exon 9 skipping, reduced total CUL3 mRNA and protein abundance. No change in WNK4, pOSR1, and pNCC abundance in kidney. Normal electrolyte level and blood pressure
Murthy et al.(2016) [52]	*Cul3*^+/Δ403–459^ Schumacher et al	No differences in ROMK immunostaining or distribution in the medulla of *Cul3*^+/Δ403–459^ mouse kidney compared to littermate *Cul3*^+/+^
Agbor et al.(2016) [54]	*S-Cul3Δ9* (Smooth muscle–specific CUL3Δ9 expression)	Undetectable CUL3Δ9 in aortas. Reduced abundance of WT-CUL3, increased RhoA abundance and Rho kinase (ROCK) activity. Increased systolic blood pressure (SBP). Increased collagen deposition, and vascular stiffness in response to angiotensin II
Ferdaus et al.(2017) [49]	*Cul3*^+/-^ & *Cul3*^+/-^^/Δ9^ (Renal epithelia specific *Cul3* deletion and CUL3Δ9 transgene expression)	Reduced abundance of WT-CUL3 in *Cul3*^+/-^ & *Cul3*^+/-/Δ9^.Undetectable CUL3Δ9 in *Cul3*^+/-/Δ9^. Increased abundance of WNK4, pNCC, and pNKCC2 in *Cul3*^+/-/Δ9^ but no change in *Cul3*^+/-^. No change in KLHL3 abundance along DCT. Increased plasma [K^+^] and systolic blood pressure in *Cul3*^+/Δ9^ compared to *Cul3*^+/+^ and *Cul3*^+/-^
Yoshida et al.(2018) [55]	*Cul3*^+/Δ403–459^	Increased abundance of WNK4, WNK1, pSPAK, pOSR1, and NCC in *Cul3*^+/Δ403–459^ kidney lysates. Hyperkalemia and metabolic acidosis in *Cul3*^+/Δ403–459^ compared to WT mice. Reduced abundance of KLHL3 in *Cul3*^+/Δ403–459^ kidney and brain lysates, but no change in KLHL2, KEAP1 and KLHL16 protein abundance
Abdel-Khalek et al.2019 [56]	pgk-*Cul3*^+*/*Δ9^ (Ubiquitous CUL3 expression) SM22*-Cul3*^+/VSMΔ9^ (Vascular smooth muscle specific CUL3 expression)	Reduced abundance of total CUL3 in pgk-*Cul3Δ9* kidneys and in pgk-*Cul3Δ9* and SM22-*Cul3Δ9* aorta. Increased abundance of WNK4, pSPAK, pOSR1, pNCC and Hyperkalemia, hyperchloremia and increased aldosterone-to-creatinine ratio in pgk-*Cul3Δ9* mice, but not SM22-*Cul3Δ9* mice. No change in the KLHL3 abundance in the kidneys of pgk-*Cul3Δ9* mice. Increased SBP in in pgk-*Cul3Δ9*. Increased both systolic and diastolic blood pressure in SM22-*Cul3Δ9* mice. Increased abundance of RhoA and ROCK substrate pMYPT1 in SM22-Cul3Δ9 mice aorta
Agbor et al.2019 [57]	S-*Cul3*^*−/−*^Smooth muscle–specific deletion of CUL3(S-CUL3KO)	Severe hypertension and increased arterial stiffness. Decreased expression of the nitric oxide (NO) receptor soluble guanylate cyclase (sGC). Reduced NO responsiveness and reduced cGMP activity in vascular smooth muscle
Maeoka et al.2022 [50]	*Cul3*^+/-/Δ9^ *Cul3*^+/-^ *Klhl3*^+/-^	Reduced abundance of KLHL3 in *Cul3*^+/-Δ9^ mice. Combined *Cul3*^+/-^ *Klhl3*^+/-^ mice showed increased abundance of WNK4, pSPAK, pNCC, and FHHt-like phenotype (higher plasma [K^+^], salt-sensitive hypertension, normal plasma [Cl^−^], no metabolic acidosis)

### Mechanisms of electrolyte imbalance and hypertension in *CUL3*-induced FHHt

NCC overactivation is the central physiological mechanism behind the electrolyte imbalance in patients with FHHt. Due to the close relationship between NCC activation, electrolyte homeostasis, and blood pressure regulation, FHHt and Gitelman syndrome also serve as conduits for a greater understanding of NCC pathophysiology. FHHt patients with* Cul3* variants often develop the most severe and earliest manifestations of FHHt [14]. However, there has been no direct functional analysis to confirm increased NCC activation in samples of FHHt patients with CUL3Δ403–459. Recently our group showed increased SPAK/OSR1 phosphorylation as well as increased total and phosphorylated NCC in the urinary exosomes of a patient with a novel CUL3 variant (Δ474–477) [21]. This study provides the first direct evidence of activation of the SPAK-OSR1-NCC cascade in an FHHt patient.

**Fig. 2:**
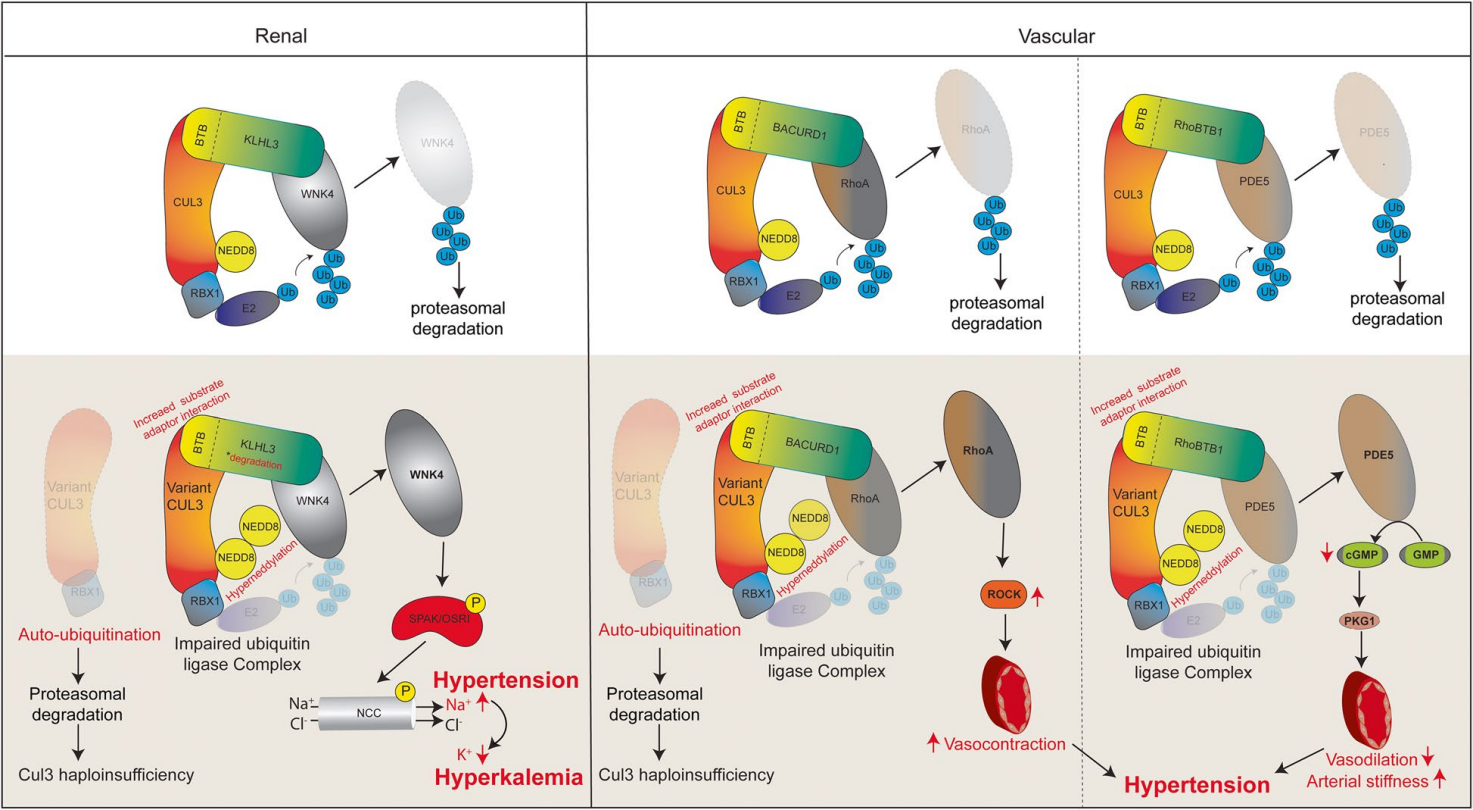
The FHHt-causing CUL3 variant (Δ403–459 or Δ474–477*)* dysregulates electrolyte balance and blood pressure using different pathophysiological mechanisms. The enhanced auto-ubiquitination-induced proteasomal degradation of variant CUL3 reduces total CUL3 levels that produce functional haploinsufficiency. The remaining variant CUL3 exhibits hyperneddylation and increased binding with the substrate-adaptor, resulting in catalytically impaired ubiquitin ligase complexes. As a result, the increased accumulation of substrates disrupts normal physiological processes. (Left) In the renal system, these mechanisms lead to the accumulation of WNK4, a substrate of the CUL3-KLHL3 ligase complex. WNK4 hyperactivates NaCl-cotransporter (NCC) by SPAK/OSR1-mediated phosphorylation, resulting in increased Na^+^ reabsorption in the distal convoluted tubule (DCT), which contributes to hypertension and affects downstream K^+^ excretion, leading to hyperkalemia in FHHt. *Some studies have showed that enhanced degradation of KLHL3 by CUL3Δ403–459 also contributes to WNK4 accumulation[50, 55]. However, this mechanism remains controversial. (Right) In the vascular system, the combination of enhanced auto-ubiquitination of CUL3Δ403–459 and the formation of catalytically impaired CUL3-BACURD1 and CUL3-RhoBTB1 ligase complexes cause the accumulation of RhoA and PDE5 substrates, respectively. Increased RhoA/ROCK kinase signaling enhances vasoconstriction, while increased PDE5 reduces the bioavailability of cGMP, leading to reduced vasodilation and increased arterial stiffness. The combination of these actions may collectively contribute to hypertension in FHHt

As such, despite no evidence from samples of patients harboring CUL3Δ403–459, a rough consensus pathophysiological mechanism behind the electrolyte and metabolic abnormalities in FHHt has been reached through a combination of studies in cultured cells, mice, and human CUL3Δ474-477 samples. Overactivation of the WNKs and SPAK/OSR1 phosphorylation cascade increases pNCC (Fig. 2). This increased cascade activity ultimately decreases Na^+^ in the distal lumen and cortical collecting ducts of the nephron while increasing Na^+^ reabsorption. Decreased Na^+^ delivery to the CCD decreases sodium flux through ENaC channels, secondarily impairing CCD electrochemical gradients. This decreases K^+^ excretion through CCD ROMK channels, ultimately inducing hyperkalemia [37] (Fig. 2). Also, as a result of the increased Na^+^ and Cl^−^ reabsorption, the macula densa activation of the renin–angiotensin–aldosterone system (RAAS) is diminished leading to decreased H^+^ excretion and non-anion gap metabolic acidosis [58]. Moreover, hyperkalemia induced by lower Na^+^/K^+^ exchange in the collecting duct lowers proximal tubule ammonia generation and collecting duct ammonia transport. The resulting decrease in ammonia excretion could also be potentially responsible for metabolic acidosis in FHHt [59].

However, the underlying molecular mechanism of WNK accumulation due to variant CUL3 has not reached a consensus and is subject to much debate. Our study suggests that increased accumulation of WNK4 in an FHHt patient carrying the CUL3Δ474-477 is the net effect of reduced total CUL3 levels due to increased autoubiquitination and formation of catalytically impaired CUL3 ligase complexes [21]. Transfection studies showed that WNK4 accumulation resulted from an increased ability of the canonical CUL3 variant (Δ403–459) to ubiquitinate and degrade substrate-adaptor KLHL3 [51, 52]. The enhanced KLHL3 ubiquitination is likely a result of increased structural flexibility of CUL3Δ403–459 [52], which also allows auto-ubiquitination and subsequent proteasomal degradation of the active CUL3Δ403–459 ligase itself.

Nevertheless, there continues to be controversy surrounding the mechanism behind CUL3-induced KLHL3 regulation in FHHt, specifically in terms of transfection experiments vs mice and human studies. The major weakness of transfection studies is that they examine the effect of overexpressed CUL3Δ403–459 in cells, which expresses ectopic CUL3 at a much higher level compared to endogenous CUL3. While this approach might accurately demonstrate the higher ubiquitin ligase activity of CUL3Δ403–459 in relation to KLHL3 exclusively, whether these findings represent total CRL3 activity in the context of heterozygous CUL3Δ403–459 (*CUL3*^+/Δ403–459^) in FHHt patients is questionable. While KLHL3 is abundantly expressed in kidney DCT cells [51], some studies performed in CUL3Δ403–459 heterozygous mice showed no change [49, 52, 56] and others showed reduced [50, 55] KLHL3 abundance compared to wild-type mice; in both cases, there was an increased accumulation of WNK4. While the studies showing no difference used an antibody validated in transfected cells, the studies showing lower KLHL3 used an antibody validated in kidney lysates from *Klhl3* knockout mice. As such, further investigation is required to resolve this issue. Using an anti-KLHL3 antibody (Proteintech;16951–1-AP), which was validated in *Klhl3* knockout mice [45], there was no significant change in KLHL3 abundance in cells derived from an FHHt patient harboring a heterozygous CUL3Δ474-477 variant [21]. This suggests that WNK4 accumulation in cells harboring heterozygous CUL3Δ474-477 is likely not due to reduced KLHL3 abundance, but rather due to changes in the abundance and activity of CRL3 ligase complexes. In support of this, studies in CUL3Δ403–459 mice and in CUL3Δ474-477 patient-derived cells showed that FHHt-causing *CUL3* variants lead to enhanced autoubiquitination, resulting in a reduced abundance of total CUL3 [21, 52]. This would result in the haploinsufficiency of functional WT-CUL3, which in combination with catalytically impaired CUL3 ligase complexes, is likely responsible for increasing the total abundance of substrate WNK4. However, a complete loss of *Cul3* exclusively in the nephron led to widespread kidney dysfunction, including salt-sensitive hypotension [51], while a partial loss of *Cul3* (*Cul3* heterozygous) did not induce disease symptoms [49]. These findings suggest that the presence of a dominant allele harboring the *CUL3 *variant is essential for inducing FHHt-specific symptoms. Recent studies in combined *Cul3* and *Klhl3* heterozygous mice (*Cul3*^+/-^ /*Klhl3*^+/-^) showed an FHHt-like phenotype, suggesting that in addition to WT-CUL3 haploinsufficiency, reduction in the KLHL3 abundance contributes to the disease pathology [50]. However, in the absence of clear consensus about the KLHL3 abundance in CUL3Δ403–459 heterozygous mice [50, 52, 55, 56], the contribution of reduced KLHL3 abundance in the renal mechanism of the disease remains controversial.

The salient features of variant CUL3 are hyperneddylation (due to interference in the CSN binding site) and an increased ability to interact with different substrate adaptors (such as KLHL3 for WNK4 degradation) [21, 51, 60]. The change in binding to the substrate may also alter the dimerization of active CRL3 ligase [61]. The resulting variant/WT CUL3 heterodimers may be unstable [60], sequester adaptors and restrict their ability to efficiently transfer ubiquitin to the substrate, leading to the formation of catalytically impaired ubiquitin ligation assemblies. Thus, the combination of haploinsufficient WT-CRL3 (due to enhanced autoubiquitination) and the formation of functionally impaired ubiquitin ligation assemblies likely results in increased substrate accumulation due to FHHt-causing CUL3 variants.

Thiazide diuretics, which function by deactivating the NCC, directly resolve electrolyte abnormalities and lower high blood pressure in FHHt patients, providing further evidence of NCC’s role in FHHt [21]. However, the exact mechanism by which thiazides mitigate hypertension and electrolyte abnormalities broadly remains a debate. Multiple mechanisms have been proposed regarding the direct and indirect effects of thiazide diuretics on arterial vasodilation [62]. Others propose that ion transport and extracellular fluid overload are the main pathophysiologies behind thiazide-regulated hypertension [63]. As mentioned previously, Harris et al. experimentally demonstrated that the correction of hyperkalemia by thiazide administration also corrected the metabolic acidosis by increasing ammonia excretion and normalizing the abnormal expression of key enzymes in ammonia metabolism [59].

Thus, while the WNK-SPAK-NCC pathway is likely pathogenic for the hyperkalemia and metabolic acidosis seen in FHHt patients, the exact mechanism behind the pediatric onset of hypertension in *CUL3*-induced FHHt has yet to be solidified. Sigmund et al.proposed potential mechanisms behind hypertension in FHHt due to CUL3Δ403-459, all of which revolve around increased arterial stiffness through RhoA/Rho kinase signaling. In support of these findings, Pelham et al.first showed a reduction of CUL3 binding protein RhoBTB1 [64] in mice expressing a human hypertension-causing variant in peroxisome proliferator-activated receptor γ (PPARγP467L) [65]. This study showed that siRNA-mediated knockdown of *Cul3* in the wild-type primary rat aortic smooth muscle cells leads to an increased abundance of RhoBTB1 and RhoA as well as increased arterial pressure in vivo, suggesting that CUL3 regulates the vascular function and arterial pressure [65]. Later, this same group showed increased interaction of CUL3Δ403-459 with RhoBTB1 and Rho A substrate adaptor, BACURD1 (BTB/POZ domain-containing adapter for CUL3-mediated RhoA degradation protein 1, also known as KCTD13), as well as defective RhoA ubiquitination. This is further evidence of increased RhoA abundance in the presence of CUL3Δ403-459 [60]. Subsequently, Agbor et al.established a vascular phenotype of the CUL3Δ403-459 mice in concordance with the hypertension seen in FHHt. This study showed that transgenic mice selectively expressing CUL3Δ403-459 in smooth muscle cells (S-CUL3Δ9) exhibited RhoA accumulation and increased arterial blood pressure relative to wild type after equivalent doses of angiotensin II were administered as artificial stressors [54].

Interestingly, conditional loss of *Cul3* in primary aortic smooth muscle cells in mice led to an even greater magnitude of hypertension compared to transgenic CUL3Δ403-459 mice [57]. The loss of *Cul3* also caused impaired ubiquitination and increased accumulation of PDE5 (Phosphodiesterase 5A), a negative regulator of the Nitric Oxide (NO)-soluble guanylyl cyclase (sGC)-cGMP pathway. This resulted in decreased production of cGMP, and diminished vasodilation ultimately leading to vascular dysfunction, increased arterial stiffness, and hypertension in mice [57]. Of note, RhoBTB1 serves as a CRL3 substrate adaptor for the ubiquitination of PDE5, further suggesting that enhanced binding of variant CUL3 with substrate adaptors results in impaired substrate (i.e., RhoA and PDE5) ubiquitination and diminished vascular compliance in FHHt hypertension (Fig. 2). Recently, Wu et al.showed that mice selectively expressing *CUL3Δ9* (CUL3Δ403-459) in the vascular endothelium (E-*Cul3Δ9*) exhibit impaired activation of endothelial nitric oxide synthase (eNOS) due to dephosphorylation by CUL3 substrate protein phosphatase 2A (PP2A), culminating in decreased NO biogenesis and endothelial dysfunction [66].

Interestingly, E-*Cul3Δ9* mice were also susceptible to salt-induced hypertension, likely caused by defective endothelium-dependent vasodilation because of decreased NO bioavailability [67]. By comparing the effects of *Cul3Δ9* in both distal nephron and vascular smooth muscle, Abdel-Khalek et al. also showed that the *Cul3* variant induces hypertension through independent renal and vascular mechanisms. The authors showed that knock-in mice bearing *Cul3Δ9* only in vascular smooth muscle (*SM22-Cul3Δ9*) exhibited both systolic and diastolic hypertension. This finding implies that vasoconstriction induced in renal blood vessels in *SM22-Cul3Δ9* mice might lead to diastolic hypertension. However, no effect on renal transport and activation of the WNK-NCC pathway was observed in *SM22-Cul3Δ9* mice in contrast to mice ubiquitously expressing *Cul3Δ9* (*pgk-Cul3Δ9*), which showed both electrolyte imbalance and hypertension [56]. Collectively, these findings provide evidence that *CUL3*-induced hypertension in FHHt is caused by alteration of both renal and vascular mechanisms (Fig. 2). Moreover, these findings collectively provide evidence that CUL3 plays an important regulatory role in blood pressure physiology and indicate that variant CUL3 dysregulates not only substrates and BTB substrate adaptors related to the DCT but also substrates that perpetuate other CUL3 systemic diseases that manifest in CUL3 induced FHHt.

### Patients with *CUL3* variants present with classical FHHt phenotypes and other systemic disturbances

Boyden et al. first reported seventeen patients with autosomal dominant *CUL3* variants identified through exome sequencing. These variants were clustered in sites implicated in exon 9 splicing [14]. Recently Hureaux et al. reported a cohort of nineteen patients with *CUL3* variants, all leading to exon 9 skipping [17]. In both cohorts, the prevalence of de novo* CUL3* variants was extremely high. Through exome sequencing of a pediatric FHHt patient and his family, our group identified a non-canonical de novo* CUL3* variant that led to a deletion of four amino acids in exon 10 (CUL3Δ474-477) [21].

In addition to the classical phenotypic triad of hypertension, hyperkalemia, and metabolic acidosis, FHHt patients with *CUL3* variants experienced diverse systemic and early developmental disturbances like failure to thrive, malaise, and paralysis. Growth disturbances and delays are most frequently seen, as many patients presented with low birth weights, failure to thrive, and short stature [14, 17, 21, 68–70]. In the cohort reported by Hureaux et al., 71% of *CUL3* patients with exon 9 skipping displayed growth failure [17]. Other developmental disturbances were also reported in specific case reports of patients with *CUL3* variants (Table 2). Autism spectrum disorder and intellectual impairment have been seen in FHHt patients with *CUL3* variants as well [21, 70, 71]. The *CUL3* variant located in exon 10, CUL3Δ474-477, displayed developmental delays, intellectual impairment, the classic FHHt triad, as well as other systemic issues like recurrent pulmonary infections, hyperreflexia, hypotonia, and non-specific thinning and volume loss of lateral ventricular white matter.

**Table 2:** List of published FHHt-associated *CUL3* variants and clinical symptoms of the patients

Publication	*CUL3* variant (NM_003590.5)	Age	Classical FHHt findings	Other clinical findings
FHHt Case Reports				
Tsuji et al. (2013) [19]	c.1377 G > C	3 years	Hyperkalemia, hyperchloremia, metabolic acidosis, hypertension	Normal growth and development
Osawa et al. (2013) [18]	c.1207-6 T > G*	1 year	Hyperkalemia, metabolic acidosis, hypertension	Low birth weight, growth retardation at 10 months old, no developmental delay
Hollander et al.(2016) [72]	c.1377 + 1G > A	6 years	Hyperkalemia, borderline blood pressure	Hematuria
Shao et al.(2018) [73]	c.1221A > G	56 years	Hyperkalemia, hyperchloremic metabolic acidosis, hypertension	Growth impairment with short stature, hypertension before 18 years of age
Shao et al.(2018) [73]	c.1221A > G	35 years	Hyperkalemia, hyperchloremic metabolic acidosis, hypertension	Growth impairment with short stature, hypertension before 18 years of age
Shao et al.(2018) [73]	c.1221A > G	33 years	Hyperkalemia, hyperchloremic metabolic acidosis, hypertension	Growth impairment with short stature, hypertension before 18 years of age
Shao et al. (2018) [73]	c.1221A > G	12 years	Hyperkalemia, hyperchloremic metabolic acidosis, hypertension	Failure to thrive
Nakano et al.(2020) [71]	c.1312A > G	Genetic screen at 6 years	Hyperkalemia, metabolic acidosis, normal blood pressure	Autism spectrum disorder, mild mental retardation
Ostrosky-Frid et al.(2020) [68]	c.1207-26A > G	12 years	Hyperkalemia, hyperchloremia, metabolic acidosis, hypertension	None reported
Ostrosky-Frid et al.(2020) [68]	c.1207-26A > G	Not reported	Unaffected mother Mosaicism	N/A
Yavas Abali et al.(2020) [70]	NM_001257198.2; c.1395 + 4A > G	Adolescent	Hyperkalemia, hyperchloremia, metabolic acidosis, hypertension	Short stature, intellectual impairment, muscle weakness
Park et al.(2022) [74]	c.1377 + 1G > C	7 years	hypertension, metabolic acidosis, and persistent hyperkalemia	short stature, low birth weight, cleft palate, and polydactyly
Li et al.(2022) [75]	c.1207-12 T > A	2 years	Hyperkalemia, metabolic acidosis, hypertension	congestive heart failure reported at age 37 years
Chatrathi et al.(2022) [21]	c.1420_1431del12 (non-canonical *CUL3* variant)	3 years	Hyperkalemia, hyperchloremia, metabolic acidosis, hypertension	Gastroesophageal reflux disease, chronic aspiration, frequent pulmonary infections, dysmorphic facial features with triangular face and chin cleft, hypotonia, hyporeflexia, facial asymmetry, white matter thinning, short stature and thin habitus, speech dyspraxia, hypermobility of joints
FHHt Cohort with *CUL3* variants				
Boyden et al.(2012) [14] *13 variants, 17 patients*	c.1207-28 T > G c.1207-26A > G (4 patients) c.1207-12 T > G c.1207-3C > A c.1207-1G > A c.1238A > G (2 patients) c.1236G > A c.1376_1377 + 4del c.1377G > A c.1377 + 1dup c.1377 + 1G > C c.1377 + 3A > G c.1207-5 T > A	9 ± 6 years	Hyperkalemia, hyperchloremia, metabolic acidosis, hypertension	Growth impairment
Glower et al. (2014) [20]	C.1377 + 1G > T C.1377 + 1G > C c.1207–12 T > A c.1207-1G > A	2,3,11 and 21 years	Hyperkalemia, hyperchloremia, metabolic acidosis (*reported in 2 cases*) Hyperkalemia, hypertension (*reported in 2 cases*)	None reported
		(7 ± 8.8 years (median age)		
Hureaux et al.(2021) [17] *11 variants, 19 patients*	c.1207-26A > G c.1207-17_1207 10delinsAAGAT c.1207-3C > A c.1207-2A > G c.1207-1G > A c. 1207-1_1207delinsAG c.1236G > A c.1377 + 1G > A c.1377 + 2 T > C (2 patients) c.1377 + 3A > T c.1377 + 4A > G	5.5 years (Median age)	Hyperkalemia, hyperchloremia, metabolic acidosis, hypertension	Growth retardation, muscle fatigue and episodes of paralysis, Sharp T waves on ECG, developmental and behavioral disorders

Reported *CUL3* variants associated with FHHt using GenBank accession number and version of the cDNA reference sequence NM_003590.5 unless otherwise stated. *The cDNA coordinates of the variant reported (c.382–6 T > G) in the original publication [18]. Age of patients at the time of diagnosis. Clinical symptoms reported in the individual patient (case reports) and in cohort studies are further categorized as classical findings (commonly found in FHHt patients) and other clinical findings as reported in the respective publication

The developmental disturbances associated with *CUL3* variants are unique and appear to result in more severe disease and earlier onset relative to WNK1, WNK4, and KLHL3 variants. Not all *CUL3* variants cause developmental disturbances; one reported *CUL3* patient, who presented with hematuria, displayed hyperkalemia and borderline blood pressure without any other growth or developmental delays [72].

### Global changes in the CUL3 interactome network provide insights into diverse FHHt phenotypes

*CUL3* is a ubiquitously expressed gene, making the widespread pathological consequences of dominant *CUL3* variants credible. Thus far, research efforts are focused on the mechanisms behind electrolyte imbalance and hypertension associated with FHHt patients. Non-renal phenotypes clinically reported in patients are not effectively appreciated as part of the canonical FHHt presentation. This is likely due to a lack of mechanistic studies rationalizing *CUL3* variant-associated effects outside of the renal and vascular systems. Recent proteomic analyses began tackling this question by analyzing the global consequences of CUL3Δ403-459 and CUL3Δ474-477. Our group performed the first proteomic analysis using FHHt patient-derived cells and HEK-293T cells expressing epitope-tagged CUL3, CUL3Δ403-459 or CUL3Δ474-477, providing an overview of CUL3 interactome networks [21]. Recently, Kouranti et al. performed quantitative proteomic analysis using HEK-293 cells stably expressing CUL3 or CUL3Δ403-459 under the control of the inducible Tet promoter and identified interacting proteins [76].

Both proteomic analyses confirmed the reduced interactions of CUL3 variants with deneddylase COP9 signalosome (CSN) subunits and identified increased interactions with the cullin inhibitor Glomulin [21, 76] and deubiquitinating enzyme USP25 [76]. Interestingly, CAND1, which plays a regulatory role in the dynamic assembly of CRL complexes, exhibited reduced interaction with CUL3Δ403-459 [21, 76], but not with CUL3Δ474-477 [21]. The differential CAND1 binding highlights possible differences in the structural rearrangements induced by CUL3Δ403-459 and CUL3Δ474-477 that likely affect CRL3 regulation in all tissues where CUL3 is expressed. CAND1 is, therefore, less likely to provide a rationale for some of the unique patient phenotypes, thus far only reported in patients with CUL3Δ474-477, such as dysmorphic facial features and speech dyspraxia.

**Fig. 3:**
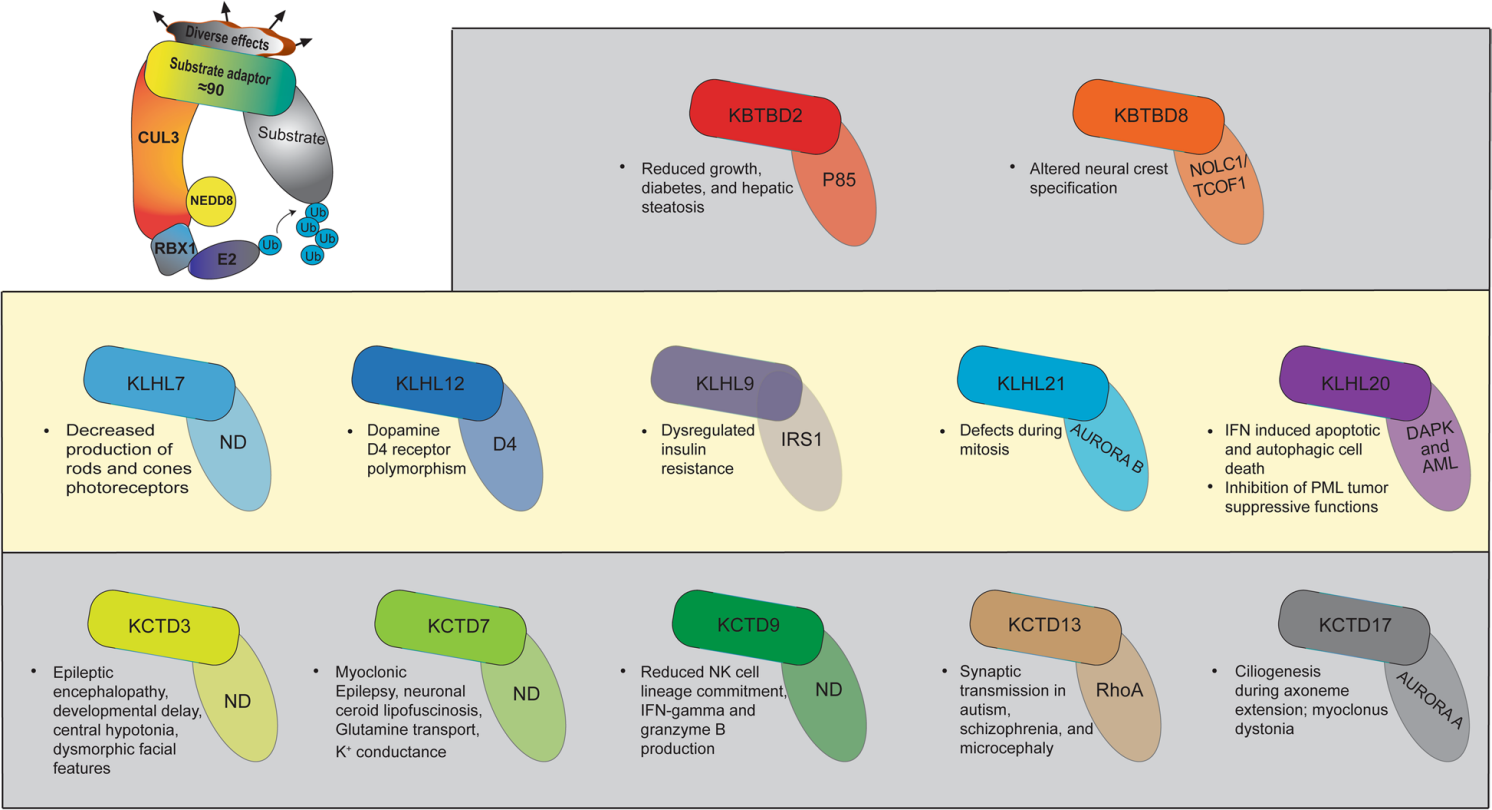
The CUL3-RING ubiquitin ligase plays a critical role in a wide array of physiological processes by ubiquitinating many different substrates, each with specific BTB substrate adaptors. The substrate adaptors that show increased binding to FHHt-causing CUL3 variants in proteomic analyses reported in Chatrathi et al.(2022) and Kouranti et al.(2022) with dysregulated physiological processes are shown. (Top) Substrate adaptors Kelch repeat and BTB domain-containing protein (KBTBD) 2 and 8 regulate growth development and neural crest cell differentiation, respectively. (Middle) Kelch-like (KLHL) substrate adaptors regulate retinal effectiveness (KLHL7), dopamine-receptor signaling (KLHL12), insulin signaling (KLHL9), mitosis (KLHL21), IFN-induced apoptosis, autophagy and tumor suppressive effect of PML (KLHL20). (Bottom) The variants found in the member of the potassium channel (K^+^) tetramerization domain (KCTD) family are associated with neurocognitive (KCTD3), neurodevelopmental (KCTD7), autism and schizophrenia (KCTD13), and movement disorders (KCTD17). *Kctd9* deficient mice exhibit impaired NK cell lineage commitment, insufficient IFN-γ, and granzyme B- production. ND; not discovered

Proteomic analyses also highlighted the enhanced binding of CUL3 variants to multiple BTB substrate adaptor proteins [21, 76], which likely results in diverse biochemical and physiological manifestations. Here we highlight the BTB adaptors that demonstrated an increase in binding with both CUL3Δ403-459 and CUL3Δ474-477 as well as BTB adaptors with possible relevance to the clinical phenotypes (Fig. 3). First, the kelch-like gene family showed consistently elevated binding to CUL3 variants. KLHL3, the BTB substrate adaptor most pertinent to the pathophysiology of FHHt, showed increased CUL3 binding. However, other Kelch-like proteins also showed increased binding to CUL3 variants like KLHL7, KLHL9, KLHL12, KLHL20, and KLHL21[21, 76]. Interestingly, a dominant negative *KLHL7* variant was found in a patient with autosomal dominant retinitis pigmentosa, which progressively decreased the production of rods and cones photoreceptors [77]. Variant KLHL7 attenuates its interaction with CUL3 and diminishes E3 ligase activity, suggesting a role for the CUL3-KLHL7 complex in the clearance of toxic substrates in photoreceptor cells [77].

The CUL3-KLHL12 complex also regulates dopamine signaling, implicated in many neurological disorders, such as attention deficit hyperactivity, Parkinson’s disease, and schizophrenia, by targeting dopamine receptor D4 for polyubiquitination [78]. Moreover, KLHL9 and KLHL21 showed some of the highest BTB adaptor binding levels to CUL3 variants relative to the wild-type. The development of distal myopathy and atrophy of distal limb muscles has been attributed to a missense variant in *KLHL9* [79]. KLHL9 forms a CRL3 complex with CUL3 and KLHL13 to target insulin receptor substrate-1 (IRS1), critical for insulin signaling [80]. KLHL21 is preferentially expressed in skeletal muscle, heart muscle, and to a lesser extent in the brain [81]. Interestingly, KLHL21, KLHL9, and KLHL13 all form distinct ligase complexes with CUL3 to mediate Aurora B ubiquitination during mitosis, likely by nonredundant mechanisms [82]. It would be interesting to explore whether increased binding of variant CUL3 to these substrate-adaptors affects the orderly progression of mitosis, which may provide some explanation for the growth impairment seen in many CUL3-induced FHHt cases. Finally, KLHL20 forms a complex with CUL3 to target both DAPK (death-associated protein kinase) and PML (promyelocytic leukemia) and is implicated in IFN-induced apoptotic cell death and autophagy. In addition, KHL20 has been reported to amplify endothelial cell migration and angiogenesis, cytoskeletal regulation, vesicular trafficking, and neural morphogenesis [83].

The function of potassium channel (K^+^) tetramerization domain (KCTD) family members to serve as substrate adaptors in the CRL3 complex for specific substrate ubiquitination is not well known. However, members of the KCTD family, specifically *KCTD3*, *KCTD9*, *KCTD13*, and *KCTD17*, have each been associated with pathological processes related to the phenotypes observed in FHHt-patients with *CUL3* variants [84]. Patients with *KCTD3* variants have presented with developmental epileptic encephalopathy, developmental delay, central hypotonia, and diverse and dysmorphic facial features. Other *KCTD3* patients presented with hydrocephalus, abnormal brain myelination, and polycystic kidney disease [85]. In addition, *Kctd9* knockout mice exhibited impaired NK cell lineage commitment, decreased IFN-gamma production and degranulation, and decreased granzyme B production [86]. The CULΔ474-477 patient had recurrent pulmonary infections that could have been the result of diminished functional KCTD9 and deficient immunological response to pulmonary pathogens neonatally. A *kctd13* deletion in mice resulted in reduced synaptic transmission due to an increase in RhoA, a CUL3-KCTD13 substrate [87]. It is possible that aberrant CUL3 and KCTD13 binding sequesters KCTD13, leading to excess RhoA and neurological development issues seen in several *CUL3* patients. Depletion of KCTD17 arrested ciliogenesis at the step of axoneme extension, which is more evidence of CUL3 and BTB adaptors affecting diverse systemic processes [88].

The Kelch repeat and BTB domain-containing (KBTBD) proteins, specifically KBTBD2 and KBTBD8, also showed increased binding to both CUL3Δ403-459 and CULΔ474-477. *Kbtbd2* knockout mice resulted in insulin-resistant diabetes, hepatic steatosis, and significantly reduced growth from 4 weeks of age to 29 weeks of age relative to wild type [89]. The growth deficiencies in *Kbtbd2* mice corroborate the developmental growth deficiencies seen in most *CUL3* variants. Sequestration of BTB adaptors due to aberrant CUL3 binding is a possible mechanistic explanation. The same is true for KBTBD8, which when translationally blocked in *Xenopus tropicalis*, led to deficient and altered differentiation and specification of neural crest cells. This presentation in vivo echoed the craniofacial pathophysiology of patients with Treacher-Collins Syndrome [90]. FHHt patients with *CUL3* variants presented with facial abnormalities like cleft palate, triangular facies, and other dysmorphic facial features; as a result, KBTBD8 could represent a source of study for these developmental abnormalities.

It is important to mention here that while CUL3 is broadly expressed, it is the relative abundance of CUL3 and BTB-adaptors (CUL3: BTB-adaptor ratio) that may ultimately determine the tissue-specific activity of CRL3 ligase. The dual ability of variant CUL3 to bind robustly with BTB-adaptors and enhance auto-ubiquitination may result in abnormal CUL3:BTB adaptor ratio and the formation of CUL3 dimers (WT-WT homodimer or WT-mutant heterodimer). This may diminish the tissue-specific function of CRL3 ligase, which provides a rationale for the diverse FHHt phenotypes. As such, because of the numerous extra-renal developmental irregularities seen in multiple *CUL3* variants, it is limiting to categorize CUL3-induced FHHt as a renal-exclusive disease. We believe it may be more effective to rename CUL3-mediated forms as “FHHt with developmental dysregulation” to better describe the extra-renal manifestations of *CUL3* variants for future studies. Despite differences in the severity of classical FHHt phenotypes (hyperkalemia and hypertension), a more detailed description of clinical phenotypes in FHHt patients with CUL3Δ403-459, may ultimately help in better defining the relative severity of both variants.

### Therapeutic prospects

Currently, thiazide diuretics are the gold standard treatment for hypertension and metabolic abnormalities in FHHt patients. The WNK-SPAK/OSR1 signaling pathway also provides multiple possible targets for therapeutic intervention (reviewed recently by Meor Azlan et al.) [91]. In the hypertension phenotype of CUL3-induced FHHt, CUL3-BACURD1-RhoA/ROCK and CUL3-RhoBTB1-PED5-cGMP signaling pathways are associated with increased vasoconstriction and decreased vasodilation, respectively [61, 62]. As such, targeting these pathways could conceivably result in decreased vascular stiffness, a major component in hypertension pathophysiology and cardiovascular disease.

Another alternative therapeutic option for FHHt is to inhibit/reduce CUL3 hyperneddylation. The neddylation inhibitor MLN4924 potently inhibits cullin neddylation and is currently in multiple phase I/II clinical trials owing to its anti-tumor property (https://clinicaltrials.gov). However, targeting the neddylation broadly may not be a feasible strategy, as it may lead to non-specific effects or cytotoxicity in tissues where FHHt symptoms are not manifested. Similarly, targeting deneddylation to increase neddylation and subsequently, the activity of wild-type CUL3 (since *CUL3* variants are heterozygous) may promote over-activation and self-ubiquitination.

Recently Liu et al. showed that skipping of exon 9 in *CUL3* caused by the c.1221A > G and c.1236G > A variants can be rescued by introducing a synonymous variant, c.1224A > G(A18G) [92]. Given the recent success of gene therapy approaches to treat monogenic disorders, increasing exon inclusion by ‘splice-modulation’ or splice-correction’ therapies offers an attractive strategy to correct splicing defects. However, several key technical challenges that need to be resolved before translating this therapeutic approach to the clinic. One of the challenges is developing an effective antisense oligonucleotide or pharmacological reagent that can restore the skipping of exon 9 caused by different *CUL3* genetic variants. Another challenge is delivering these reagents to all affected tissues. Other non-genomic methods for targeting CUL3 activation could still be developed, but identification of more non-canonical FHHt disease variants in *CUL3* would help efforts to design a clearer therapeutic strategy.

## Conclusions and outstanding questions

The recent identification of a non-canonical *CUL3* variant causing FHHt, coupled with proteomic analyses of variant interactome networks, has helped to further define the role of CUL3 in FHHt symptomology. Given the vital role of CRL3 ligases in multiple cellular processes, dysregulation of these processes likely explains the multiple phenotypic outcomes in FHHt patients. This also constitutes an expanding field of great interest to identify other potentially dysregulated CRL3 complexes that operate outside of renal and vascular systems using mouse models and FHHt patient-derived cells to study pathophysiology.

However, key outstanding questions remain. The validation of WNK-SPAK/OSR1-NCC signaling activation, CUL3 hyperneddylation, levels of CUL3, and substrate adaptors in cells/tissues derived from FHHt patients harboring the CUL3Δ403–459 variant are required to strengthen our mechanistic understanding. It is currently not known if the differences in FHHt clinical phenotypes outside of the renovascular axis are due to the differential effects of *CUL3* variants on substrate-adaptor binding. Moreover, given that CUL3 can bind many substrate adaptors, we do not know what molecular mechanisms cause growth impairment, a common clinical feature of many FHHt patients. Finally, would targeting CUL3 as a therapeutic intervention for FHHt be feasible? Given the ubiquitous effects of CUL3 on a wide array of disease processes, globally targeting CUL3 would likely induce adverse systemic effects depending on the substrate adaptor and substrate affected. Regardless, a greater understanding of the biochemical mechanisms behind CUL3 pathophysiology will make the discovery of new therapies more likely.

## Data Availability

Not applicable.

## Abbreviations

4HB: 4-Helix bundle
ATP: Adenosine triphosphate
BTB: Broad-complex, Tramtrack, Bric-a-brac
CAND1: Cullin-associated NEDD8-dissociated protein 1
COP9: Constitutive photomorphogenesis 9
CRL3: Cullin-RING ligase 3
CSN: Constitutive photomorphogenesis 9 signalosome
CUL3: Cullin 3
cGMP: Cyclic guanosine 3′,5′-monophosphate
DCT: Distal convoluted tubule
eNOS: Endothelial NO synthase
FHHt: Familial hyperkalemic hypertension
KLHL3: Kelch-like-family-member-3
KS-WNK1: Kidney-specific WNK1
NCC: NaCl cotransporter
NEDD8: Neural precursor cell expressed developmentally down-regulated protein 8
NO: Nitric oxide
OSR1: Oxidative stress response kinase-1
PDE5: Phosphodiesterase 5
pMYPT1: Phospho myosin phosphatase target subunit 1
PKG1: CGMP-dependent protein kinase 1
PP2A: Protein phosphatase 2A
RBX1: RING box protein 1
RhoA: Ras homolog gene family member A
RING: Really interesting new gene
ROCK: Rho-associated protein kinase
SBP: Systolic blood pressure
sGC: Soluble guanylyl cyclase
SPAK: Ste20p-related proline alanine-rich kinase
uEV: Urinary extracellular vesicles
WNK: With-no-lysine (K)
WT: Wild type
